# Exploring the Association between Serum B Vitamins, Homocysteine and Mental Disorders: Insights from Mendelian Randomization

**DOI:** 10.3390/nu16131986

**Published:** 2024-06-21

**Authors:** Yiming Hu, Miao Yu, Yaqiang Wang, Haotian Wu, Xueqing Yang, Xiangxin Chen, Jing Wu

**Affiliations:** 1 National Center for Chronic and Noncommunicable Disease Control and Prevention, Chinese Center for Disease Control and Prevention, Beijing 100050, China; 2 School of Public Health, Inner Mongolia Medical University, Hohhot 010107, China; 3 School of Public Health, China Medical University, Shenyang 110122, China; 4 School of Public Health, Baotou Medical College, Baotou 014010, China

**Keywords:** mental disorders, B vitamins, homocysteine, mendelian randomization

## Abstract

Previous studies show that B vitamins and homocysteine (Hcy) may be associated with mental disorders, but the accurate causal relationship remains unclear. This study aimed to elucidate the potential causal relationship of serum B vitamins and Hcy levels with five common mental disorders through a two-sample Mendelian randomization (MR) study. In this MR analysis, 50 single-nucleotide polymorphisms (SNPs)—13 related to folate, 17 to vitamin B6, 8 to vitamin B12 and 12 to Hcy—were obtained from a large-scale Genome-Wide Association Studies (GWAS) database and employed as instrumental variables (IVs). The MR analyses were conducted using the inverse variance weighted (IVW), weighted median (WM), MR-Egger methods and sensitivity analyses were further performed to test the robustness. This MR study found a suggestive causal relationships between serum vitamin B12 levels and the risk of anxiety disorders (odds ratio (OR): 1.34, 95% confidence interval (CI): 1.01–1.78, *p* = 0.046) and bipolar affective disorders (OR: 1.85, 95% CI: 1.16–2.96, *p* = 0.010). However, folate, vitamin B6 and Hcy levels may not be causally associated with the risk of mental disorders. In conclusion, this study reveals that elevated serum vitamin B12 levels might suggestively increase the risk of anxiety and bipolar affective disorders, even though horizontal pleiotropy cannot be completely eliminated. The potential implications of our results warrant validation in larger GWAS based on diverse populations.

## 1. Introduction

Mental disorders involve clinically significant disruptions in cognition, emotional regulation or behavior [1,2], often causing distress or impairment, and mainly include depression, anxiety disorders, bipolar affective disorders, obsessive–compulsive disorder (OCD) and schizophrenia [3]. They are highly prevalent, affecting approximately 970 million people worldwide, or about 1 in every 8 individuals [3]. Mental disorders have a considerable impact on the lives of patients and their families, representing a significant public health concern globally and resulting in substantial medical burden and economic loss [4]. According to GBD 2019, mental disorders remain among the top ten leading causes of burden worldwide [5]. Currently, pharmacotherapy and psychotherapy are common and primary treatments. However, poor patient compliance and bias against psychological interventions result in treatment not meeting expectations [6]. Therefore, it is crucial to explore preventive and therapeutic strategies for mental disorders.

The B vitamins, including folate (vitamin B9) and vitamins B6 and B12, play essential roles as cofactors in single-carbon transfer (methylation) reactions, vital for synthesizing monoamine neurotransmitters, phospholipids and nucleotides [7,8]. Deficiency in any of these B vitamins can elevate blood levels of total homocysteine (tHcy), which is associated with an increased risk of mental illnesses such as cognitive impairment and emotional disorders [9], attributed to the neurotoxic effects of Hcy [10]. Previous research indicates that elevated Hcy levels and low folate concentrations are correlated with mental disorders [11,12], including depression [13,14,15], anxiety [16], bipolar disorder [17,18], OCD [19,20] and schizophrenia [21]. However, conclusions from observational studies regarding Hcy, B vitamins and mental disorders are inconsistent [22,23], and the precise pathogenesis among them remains unclear. Traditional observational studies may be subject to potential biases such as confounders or reverse causation [24], which can impede the accurate determination of causal associations between Hcy, B vitamins and mental disorders. Consequently, the causal role of Hcy and B vitamins in the development of mental disorders remains uncertain.

Mendelian randomization (MR) stands as a crucial epidemiological statistical method employing genetic instrumental variables (IVs) derived from large-scale Genome-Wide Association Studies (GWAS) to investigate the genetic causal associations between exposures and outcomes [25,26]. For instance, if exposure such as folate causally impacts an outcome like depression, then a variant influencing folate should theoretically affect depression proportionally. Previous studies have explored the causal relationship between serum Hcy, vitamin B and autism spectrum disorder (ASD) using the MR method, suggesting that elevated serum vitamin B12 levels might increase the risk of ASD [27]. However, to our knowledge, no studies have conducted MR analyses for Hcy, B vitamins and other mental disorders. Hence, further research is warranted to delve into the causality between mental disorders and these nutrients.

In this study, we utilized the summary statistics from large-scale GWAS to employ a two-sample MR approach in order to evaluate the causal effect between serum levels of folate, vitamin B6, B12, and Hcy and five mental disorders, namely, depression, anxiety, bipolar disorders, OCD and schizophrenia.

## 2. Materials and Methods

### 2.1. Study Design

**Figure 1:**
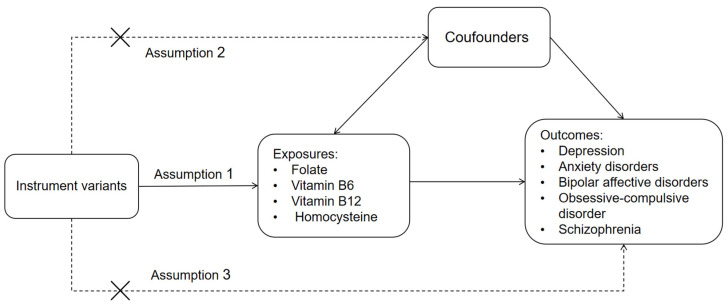
An overview of the study design.

This study adhered to the Strengthening the Reporting of Observational Studies in Epidemiology using Mendelian Randomization (STROBE-MR) guidelines [28]. The MR study was based on three fundamental assumptions [25]: (1) IVs are correlated with exposure (serum levels of folate, vitamin B6, vitamin B12 and Hcy); (2) IVs are not associated with any confounders that influence the exposure–outcome (mental disorders) relationship; and (3) IVs solely influence the outcome through exposure. An overview of the study design is illustrated in Figure 1.

### 2.2. Data Sources

GWAS summary-level data for serum levels of folate, vitamin B6 and vitamin B12 were obtained from the UK Biobank Consortium, which includes 64,979 European populations. These data are accessible through the IEU Open GWAS Project database. Additionally, GWAS data related to Hcy were acquired from a meta-analysis comprising 10 studies, with a collective sample size of 44,147 individuals of European ancestry with measured Hcy concentrations in blood [29]. As for the outcome data, summary statistics for mental disorders were extracted from the December 2023 release of the FinnGen database on GWAS data sources (R10). This encompassed depression (47,696 cases and 359,290 controls), anxiety disorders (18,903 cases and 368,054 controls), bipolar affective disorders (7569 cases and 359,290 controls), OCD (2175 cases and 368,054 controls) and schizophrenia (6708 cases and 398,386 controls). Table 1 provides detailed information on the GWAS summary-level data on exposures and outcomes analyzed in this MR study. All data analyzed herein were obtained from publicly available databases, where ethical approval was secured for each cohort and informed consent was obtained from all participants prior to their involvement.

### 2.3. Selection of Instrumental Variables

IVs were selected according to the following criteria: (1) SNPs strongly associated with vitamin B which met genome-wide significance (*p* < 5 × 10^−8^). However, due to limited number of SNPs associated with folate, vitamin B6 and vitamin B12, we screened SNPs with a more relaxed threshold (*p* < 5 × 10^−6^) for the purpose of identifying sufficient candidate instruments [27]. (2) SNPs exhibiting independence, determined through linkage disequilibrium (LD) (r^2^ < 0.001, kb = 10,000), were considered. (3) SNPs with minor allele frequencies (MAFs) ≤ 0.01 were excluded. (4) Ambiguous SNPs, characterized by non-concordant alleles and those that were palindromic with intermediate allele frequencies, were excluded during the harmonization of the exposure and outcome datasets. F-statistics were employed to evaluate weak instrumental variable bias in MR analysis, with the computation detailed in Supplementary Table S1. All IVs exhibited an F-statistic greater than 20, satisfying the assumption of F > 10 for MR analyses, indicating that the genetic variants utilized were robust IVs [30].

Lastly, 13 SNPs for folate, 17 SNPs for vitamin B6, 8 SNPs for vitamin B12 and 12 SNPs for homocysteine were selected for MR analyses. Summary statistics of the selected SNPs are provided in Table S2.

### 2.4. Statistical Analysis

We employed various MR approaches to ascertain MR estimates, including the inverse variance weighted (IVW), weighted median (WM) and MR-Egger methods. The rationale behind utilizing multiple approaches lies in their distinct underlying assumptions concerning horizontal pleiotropy. The IVW method, operating under a multiplicative random-effects model, served as the primary statistical tool. This method assumes that instruments can influence the outcome solely through the exposure of interest, without involvement in any alternative pathway [31]. Additionally, we employed the weighted median and MR-Egger methods to complement IVW estimates, as these methods offer more robust estimates across a broader range of scenarios, albeit with reduced efficiency, resulting in wider confidence intervals. The weighted median model generates consistent causal estimates under the assumption that more than half of the weights are derived from valid SNPs [32]. MR-Egger regression can detect horizontal pleiotropy through the *p*-value for its intercept and can provide estimates after correcting for pleiotropic effects, assuming instrument strength independent of direct effect [33].

Sensitivity analysis plays a crucial role in MR studies for detecting underlying pleiotropy, as heterogeneity in MR estimates can significantly impact their validity. To gauge the extent of heterogeneity, we employed Cochran’s Q statistic [34]. Additionally, the intercept derived from MR-Egger regression served as an indicator for directional pleiotropy (a significance level of *p* < 0.05) [33]. We also utilized MR-Pleiotropy Residual Sum and Outlier methods (MR-PRESSO) to assess and correct for horizontal pleiotropy [35]. Furthermore, leave-one-out analysis was conducted to assess whether the MR estimate was influenced or biased by any individual SNP.

We applied Bonferroni correction to mitigate the impact of multiple comparisons across four exposures and five outcomes, resulting in a significance threshold of α = 0.05/(4 × 5) = 0.0025 [36]. The statistical tests were two-sided, with a *p*-value < 0.0025 considered conservatively significant. *p*-values exceeding the Bonferroni-corrected threshold but falling below the conventional significance level (i.e., <0.05) were interpreted as suggestive evidence for a potential causal association [37]. All analyses were performed in R (version 4.3.1) using the “TwoSampleMR” and “MRPRESSO”.

## 3. Results

### 3.1. Mendelian Randomization Estimates

**Figure 2:**
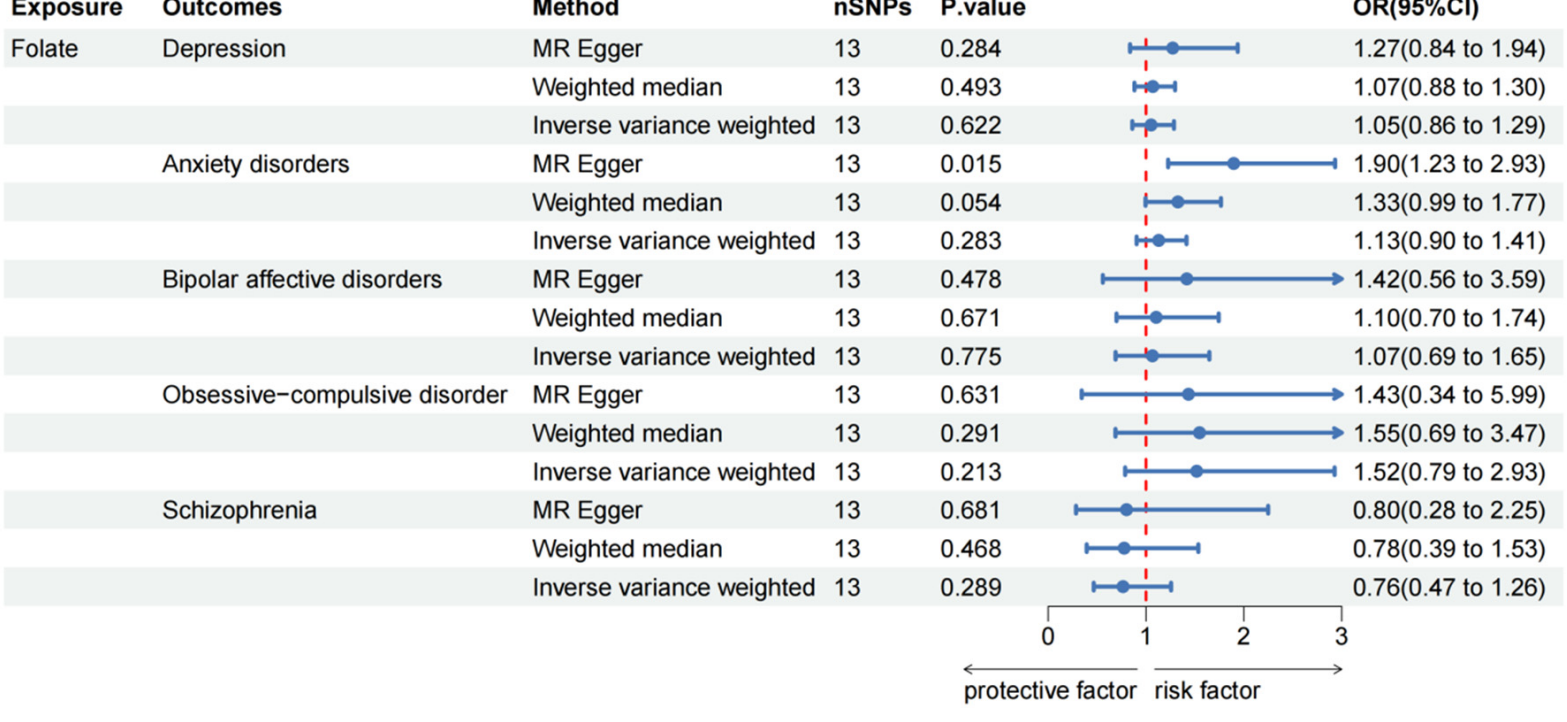
Causal effect of folate on mental disorders in MR analyses. SNP: single nucleotide polymorphisms; OR: odds ratio; CI: confidence interval; *p* value: *p* value of the causal estimate.

Figure 2 displays the results of MR analyses exploring the association between serum folate levels and the risk of various mental disorders. The findings from this study do not provide conclusive evidence of a causal relationship between serum folate levels and the risk of these five mental disorders. Specifically, no significant association between serum folate levels and the risk of anxiety disorders was detected using the IVW method (OR: 1.13, 95% CI: 0.90–1.41, *p* = 0.283). However, the MR-Egger method found a suggestive association (OR: 1.90, 95% CI: 1.23–2.93, *p* = 0.015), while the WM method closely approached suggestive statistical significance (OR: 1.33, 95% CI: 0.99–1.77, *p* = 0.054).

**Figure 3:**
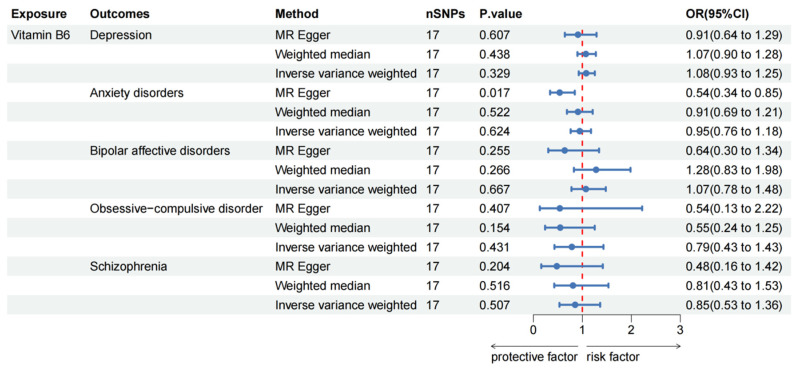
Causal effect of vitamin B6 on mental disorders in MR analyses. SNP: single nucleotide polymorphisms; OR: odds ratio; CI: confidence interval; *p* value: *p* value of the causal estimate.

A suggestively negative association between the genetically predicted vitamin B6 concentrations and risk of anxiety disorders was observed through the MR Egger method (OR: 0.54, 95% CI: 0.34–0.85, *p* = 0.017); however, other methods did not find such an association. Additionally, our MR analyses revealed no significant association between vitamin B6 and the other four mental disorders (all *p* > 0.05; Figure 3).

**Figure 4:**
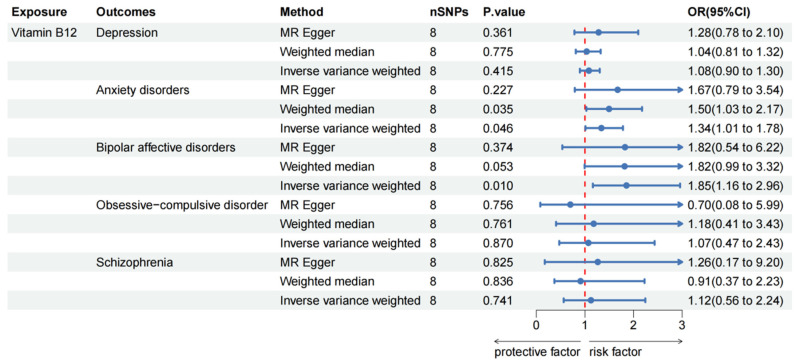
Causal effect of vitamin B12 on mental disorders in MR analyses. SNP: single nucleotide polymorphisms; OR: odds ratio; CI: confidence interval; *p* value: *p* value of the causal estimate.

For serum vitamin B12 levels, the IVW method revealed a suggestive genetic association with anxiety disorders (OR: 1.34, 95% CI: 1.01–1.78, *p* = 0.046), and a similar result was obtained using the WM method (OR: 1.50, 95% CI: 1.03–2.17, *p* = 0.035). Meanwhile, this finding was consistent with the MR-Egger method, even with *p* > 0.05. Likewise, a suggestive causal relationship between serum vitamin B12 levels and the risk of bipolar affective disorders was observed (IVW method: OR: 1.85, 95% CI: 1.16–2.96, *p* = 0.010), with the result from the WM method (OR: 1.82, 95% CI: 0.99–3.32, *p* = 0.053) closely approaching suggestive association (Figure 4 and Supplementary Figure S1). However, no potential causal association was found between serum vitamin B12 levels and depression, OCD or schizophrenia (Figure 4).

**Figure 5:**
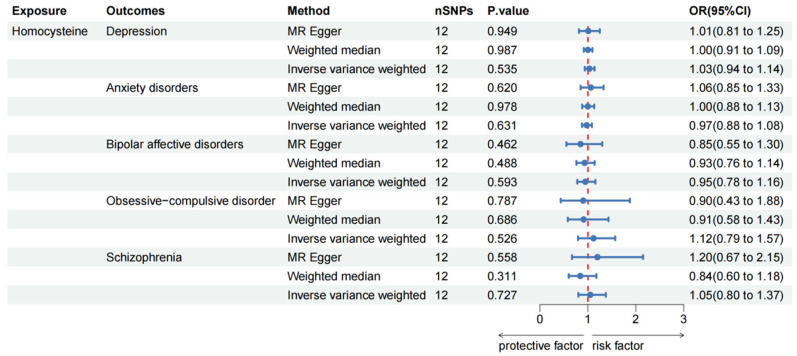
Causal effect of homocysteine on mental disorders in MR analyses. SNP: single nucleotide polymorphisms; OR: odds ratio; CI: confidence interval; *p* value: *p* value of the causal estimate.

Based on the IVW method, no significant association was observed between Hcy levels and depression (OR: 1.03, 95% CI: 0.94–1.14, *p* = 0.535), anxiety disorders (OR: 0.97, 95% CI: 0.88–1.08, *p* = 0.631), bipolar affective disorders (OR: 0.95, 95% CI: 0.78–1.16, *p* = 0.593), OCD (OR: 1.12, 95% CI: 0.79–1.57, *p* = 0.526) or schizophrenia (OR: 1.05, 95% CI: 0.80–1.37, *p* = 0.727) (Figure 5).

### 3.2. Evaluation of Mendelian Randomization Assumptions

In the sensitivity analysis, Cochrane’s Q test indicated the absence of heterogeneity in most MR analyses (Cochrane’s Q *p* > 0.05; Table S3). Even in cases where heterogeneity was observed in the analyses of folate or Hcy and depression, its impact on our results was minimal due to our utilization of the IVW method. Thus, the conclusions drawn are primarily based on our main analytical approach, the IVW method, which demonstrated no significant relationship between folate or Hcy and depression. Meanwhile, we found no evidence of horizontal pleiotropy in most association according to the MR-Egger intercept (*p* intercept > 0.05; Table S4). Finally, the MRPRESSO global test did not identify any outlier SNPs, except in the case of the association between folate, Hcy and depression (Table S5). Subsequently, we excluded these outliers and conducted a reanalysis. Supplementary Tables S6 and S7 indicate no causal association between folate or homocysteine and depression, with no evidence of heterogeneity or horizontal pleiotropy in the findings.

For the causal association analysis of vitamin B12 with anxiety and bipolar disorders, we observed no significant heterogeneity (*p* = 0.647 and *p* = 0.940, respectively; Table S3) or evidence of horizontal pleiotropy (*p* for intercept = 0.551 and 0.978, global test *p* = 0.674 and 0.937, respectively; Tables S4 and S5). These findings suggest the robustness of the MR analyses results. Furthermore, the funnel plots for the effects of vitamin B12 on the risk of anxiety and bipolar disorders are presented in Supplementary Figure S2. Additionally, the leave-one-out analysis further affirmed the stability of the MR estimates (Supplementary Figure S3). To sum up, these sensitivity analyses showed that the MR estimates were stable.

## 4. Discussion

We utilized a two-sample MR approach to thoroughly investigate the potential causal impact of B vitamins and Hcy on the prevalence of mental disorders. Our analysis revealed a suggestive causal relationship between vitamin B12 and anxiety or bipolar disorders, suggesting that elevated serum levels of vitamin B12 might increase the risk of these conditions. However, we found no clear evidence to support the genetic prediction of a causal role of serum levels of vitamin B6, folate and Hcy in influencing the risk of mental disorders.

To date, the causal relationship between folate, vitamins B6 and B12, Hcy and mental disorders remains elusive, partially due to the potential occurrence of mental disorders without hematological or neurological manifestations [38]. Previous epidemiological investigations, mostly adopting a case–control design, have struggled to establish causality due to an ambiguous chronological sequence [39,40,41]. Moreover, past observational studies have encountered challenges in mitigating bias stemming from confounding risk factors. However, in our current study, employing the MR method allowed us to confidently unveil causal relationships, thus addressing these limitations.

Folate, along with vitamins B6 and B12 and homocysteine, which are integral to one-carbon metabolism, may contribute to the pathogenesis of mental disorders [42]. The significance of one-carbon metabolism in neuropsychiatric conditions stems from its crucial role in methylation reactions essential for maintaining healthy brain tissue and function [43]. Additionally, elevated levels of homocysteine can impede the synthesis of catecholamine and non-catecholamine neurotransmitters associated with S-adenosylmethionine, thereby predisposing individuals to mental disorders like depression [44]. Moreover, homocysteine can generate neurotoxic byproducts such as homocysteine and cysteine sulfinic acid, which exert excitatory effects on *N*-methyl-D-aspartate (NMDA) receptors (excitotoxicity) and neurotoxic effects on dopaminergic neurons [45].

Previous studies have suggested that vitamin B12 deficiency may cause neuropsychiatric symptoms with clinical manifestations such as depression, anxiety and dementia [46]. Several observational studies have shown that lower vitamin B12 levels are associated with a higher risk of severe anxiety or depressive symptoms [42,47,48]. Conversely, Hadis Mozaffari et al. [49] revealed a positive association between dietary vitamin B12 intake and depression and psychological distress, albeit not anxiety, in a cross-sectional study conducted among Iranian women. Regarding bipolar disorder, Paola Mangiapane et al. [50] identified significantly lower vitamin B12 levels in bipolar patients. However, a study of elderly psychiatric inpatients found no significant difference in serum B12 levels between cognitively impaired and non-impaired patients, including those with bipolar disorder [51]. Although prior studies have accounted for confounding factors such as age [48], BMI [47] and socioeconomic status [49], they still cannot avoid the reverse causal relationship in traditional epidemiological research. Furthermore, the findings of these studies and our study are not consistent, and we believe that there are reasons for this disparity. Firstly, prior observational studies have predominantly focused on the impacts of therapeutic or low-dose vitamin B supplementation, leaving the effects of high-dose supplementation unclear. Secondly, our study emphasized serum vitamin B levels, which may not necessarily accurately reflect dietary intake. Therefore, although we derived associations from serum levels, it is essential to exercise caution when directly extrapolating these findings to dietary intake patterns and their corresponding effects.

Recent clinical studies have indicated that elevated levels of vitamin B12 are also linked to neurological disorders, cancer, and liver disease [52,53,54]. Previous studies may suggest a U-shaped relationship between vitamin B12 levels and the neuropsychiatric system, that is, wherein deficiencies and excesses of vitamin B12 may have detrimental effects. However, the potential impact of excessive vitamin B12 intake on psychiatric disorders often receives insufficient attention. Research conducted in the United States suggests that elevated serum vitamin B12 levels during pregnancy could heighten the risk of autism in offspring [55]. The findings of Sigrun Hope et al. [56] showed that the median vitamin B12 level in the neurodevelopmental disorders group was 420 pmol/L, significantly higher than that of the normal control group (316 pmol/L) and the schizophrenia group (306 pmol/L). The aforementioned studies suggest that elevated vitamin B12 levels are clinically significant in several diseases and can serve as predictors of adverse outcomes. It is worth noting that increasing vitamin B12 intake has been associated with only minimal changes in excretion, indicating that vitamin B12 levels are not solely reflective of intake [57]. This implies that genetic factors play a more significant role than environmental factors in determining vitamin B12 levels [58]. Based on the development of GWAS gene sequencing, various genetic variants have been identified that can impact serum B12 levels [59,60]. Our study utilizes genetic variants to elucidate suggestive and potential causal relationships between vitamin B12 and anxiety disorders, as well as bipolar disorder. This implies that administering vitamin B12 supplementation should be approached cautiously, with careful attention paid to dosage and monitoring of serum vitamin B12 levels. Consequently, clinicians should be cautious of using nutritional supplements in practice.

This study possesses several notable advantages. Firstly, employing a MR design enabled us to emulate a randomized controlled trial within an observational study framework. While clinical randomized controlled trials offer the highest level of evidence for establishing causality, their implementation is often prohibitively expensive and impractically challenging. However, MR studies can effectively avoid confounders and reverse causality effects based on the principle that alleles follow random assignment during gamete formation. Hence, our MR findings hold greater credibility when contrasted with previous research outcomes. Secondly, considering the elevated prevalence of mental disorders in the general population, our findings bear significant implications for the prevention, management, and treatment of such conditions, particularly concerning the utilization of vitamin B12. Our results indicate that screening for mental disorders through genetic prediction of serum homocysteine and vitamin B levels may yield limited utility. Instead, greater emphasis should be placed on addressing mental health issues stemming from environmental factors while simultaneously bolstering public health policies to enhance early prevention and timely intervention efforts.

Some possible limitations of this study need to be considered. Firstly, changes in serum trace elements may affect people with mental disorders differently by age or gender; often, women tend to be more sensitive and younger people are more susceptible to depression. Nonetheless, the absence of individual-level data within the summary statistics hindered our ability to stratify the population with mental disorders according to age and gender. Second, vitamin supplementation affects serum vitamin B levels, but the exposure data of this study did not account for whether the study population received vitamin supplementation, which could potentially influence the exposure–outcome effects. Thirdly, despite employing a range of methodologies to mitigate the impact of pleiotropy, we acknowledge that the potential bias from unknown pleiotropic effects on the results cannot be entirely eliminated. Lastly, the GWAS statistics utilized in this study were derived from European populations, thus raising a question about the generalizability of our findings to non-European populations. Consequently, future research endeavors should strive to utilize larger GWAS samples based on diverse populations to validate our conclusions comprehensively.

## 5. Conclusions

This study provides suggestive genetic evidence for the causal relationships between serum vitamin B12 concentrations and risk of anxiety and bipolar affective disorders. Specifically, genetically determined higher vitamin B12 is associated with a higher risk of anxiety and bipolar affective disorder. In the future, additional studies will be essential to ascertain the impact of B vitamins and Hcy on the initiation and advancement of mental disorders. Moreover, confirming this association necessitates large-scale randomized controlled trials and the utilization of advanced GWAS databases to ensure robustness and reliability of the findings.

## Figures and Tables

**Table 1:** Details of the GWAS summary-level data.

Trait	Sample Size	Population	Consortium	Data Accession Address
Folate	64,979	European	UKBiobank	https://gwas.mrcieu.ac.uk/
Vitamin B6	64,979	European	UKBiobank	https://gwas.mrcieu.ac.uk/
Vitamin B12	64,979	European	UKBiobank	https://gwas.mrcieu.ac.uk/
Homocysteine	44,147	European	A meta-analysis of GWAS	https://doi.org/10.3945/ajcn.112.044545
Depression	406,986	European	FinnGen	https://r10.finngen.fi/
Anxiety disorders	386,957	European	FinnGen	https://r10.finngen.fi/
Bipolar affective disorders	336,859	European	FinnGen	https://r10.finngen.fi/
Obsessive-compulsive disorder	370,229	European	FinnGen	https://r10.finngen.fi/
Schizophrenia	405,094	European	FinnGen	https://r10.finngen.fi/

## Data Availability

The GWAS data of folate, vitamin B6, vitamin B12 are accessible under application at https://www.ebi.ac.uk/gwas/ (accessed on 10 April 2024), the GWAS data of mental disorders are accessible from FinnGen database (R10) under application at https://r10.finngen.fi/ (accessed on 10 April 2024), and handling of these data are described in Materials and Methods.

## Supplementary Materials

The following supporting information can be downloaded at: https://www.mdpi.com/article/10.3390/nu16131986/s1, Table S1: STROBE-MR checklist of the present study; Table S2: Information on instrumental variables; Table S3: Heterogeneity of MR analysis for serum levels of folate, vitamin B6, vitamin B12, homocysteine and mental disorders risk; Table S4: Associations of genetic prediction of serum levels of folate, vitamin B6, vitamin B12 and homocysteine with mental disorders risk in the MR-Egger analysis. Table S5: Associations of genetic prediction of serum levels of folate, vitamin B6, vitamin B12 and homocysteine with mental disorders risk in the MR-PRESSO analysis. Table S6: Heterogeneity of MR analysis for serum levels of folate, homocysteine and depression risk after excluding the outlier SNPs. Table S7: MR analysis for serum levels of folate, homocysteine and depression risk after excluding the outlier SNPs. Figure S1: Scatter plot of the association between vitamin B12 and mental disorders; Figure S2: Funnel plot on vitamin B12 and mental disorders; Figure S3: Leave-one-out plots of vitamin B12 and mental disorders.

## References

[1] Bo Q., Dong F., Li X., Li F., Li P., Yu H., He F., Zhang G., Wang Z., Ma X. (2019). Comparison of cognitive performance in bipolar disorder, major depressive disorder, unaffected first-degree relatives, and healthy controls. Psychiatry Clin. Neurosci..

[2] Stein D.J., Palk A.C., Kendler K.S. (2021). What is a mental disorder? An exemplar-focused approach. Psychol. Med..

[3] World Health Organization Mental Disorders. https://www.who.int/news-room/fact-sheets/detail/mental-disorders.

[4] Cuijpers P., Karyotaki E., Reijnders M., Purgato M., Barbui C. (2018). Psychotherapies for depression in low- and middle-income countries: A meta-analysis. World Psychiatry.

[5] GBD 2019 Mental Disorders Collaborators (2022). Global, regional, and national burden of 12 mental disorders in 204 countries and territories, 1990–2019: A systematic analysis for the Global Burden of Disease Study 2019. Lancet Psychiatry.

[6] Asher G.N., Gerkin J., Gaynes B.N. (2017). Complementary Therapies for Mental Health Disorders. Med. Clin. N. Am..

[7] Selhub J. (2002). Folate, vitamin B12 and vitamin B6 and one carbon metabolism. J. Nutr. Health Aging.

[8] Penninx B.W., Guralnik J.M., Ferrucci L., Fried L.P., Allen R.H., Stabler S.P. (2000). Vitamin B(12) deficiency and depression in physically disabled older women: Epidemiologic evidence from the Women’s Health and Aging Study. Am. J. Psychiatry.

[9] Almeida O.P., McCaul K., Hankey G.J., Norman P., Jamrozik K., Flicker L. (2008). Homocysteine and depression in later life. Arch. Gen. Psychiatry.

[10] Ho P.I., Collins S.C., Dhitavat S., Ortiz D., Ashline D., Rogers E., Shea T.B. (2001). Homocysteine potentiates beta-amyloid neurotoxicity: Role of oxidative stress. J. Neurochem..

[11] Reynolds E. (2006). Vitamin B12, folic acid, and the nervous system. Lancet Neurol..

[12] Kronenberg G., Colla M., Endres M. (2009). Folic acid, neurodegenerative and neuropsychiatric disease. Curr. Mol. Med..

[13] Araújo J.R., Martel F., Borges N., Araújo J.M., Keating E. (2015). Folates and aging: Role in mild cognitive impairment, dementia and depression. Ageing Res. Rev..

[14] Bender A., Hagan K.E., Kingston N. (2017). The association of folate and depression: A meta-analysis. J. Psychiatr. Res..

[15] Fava M., Mischoulon D. (2009). Folate in depression: Efficacy, safety, differences in formulations, and clinical issues. J. Clin. Psychiatry.

[16] Zhao G., Ford E.S., Li C., Greenlund K.J., Croft J.B., Balluz L.S. (2011). Use of folic acid and vitamin supplementation among adults with depression and anxiety: A cross-sectional, population-based survey. Nutr. J..

[17] Hsieh Y.-C., Chou L.-S., Lin C.-H., Wu H.-C., Li D.-J., Tseng P.-T. (2019). Serum folate levels in bipolar disorder: A systematic review and meta-analysis. BMC Psychiatry.

[18] Gurholt T.P., Osnes K., Nerhus M., Jørgensen K.N., Lonning V., Berg A.O., Andreassen O.A., Melle I., Agartz I. (2018). Vitamin D, Folate and the Intracranial Volume in Schizophrenia and Bipolar Disorder and Healthy Controls. Sci. Rep..

[19] Atmaca M., Tezcan E., Kuloglu M., Kirtas O., Ustundag B. (2005). Serum folate and homocysteine levels in patients with obsessive-compulsive disorder. Psychiatry Clin. Neurosci..

[20] Türksoy N., Bilici R., Yalçıner A., Özdemir Y.Ö., Örnek I., Tufan A.E., Kara A. (2014). Vitamin B12, folate, and homocysteine levels in patients with obsessive-compulsive disorder. Neuropsychiatr. Dis. Treat..

[21] Sakuma K., Matsunaga S., Nomura I., Okuya M., Kishi T., Iwata N. (2018). Folic acid/methylfolate for the treatment of psychopathology in schizophrenia: A systematic review and meta-analysis. Psychopharmacology.

[22] Gougeon L., Payette H., A Morais J., Gaudreau P., Shatenstein B., Gray-Donald K. (2016). Intakes of folate, vitamin B6 and B12 and risk of depression in community-dwelling older adults: The Quebec Longitudinal Study on Nutrition and Aging. Eur. J. Clin. Nutr..

[23] Trincado J., Caneo C. (2018). Is augmentation with folate effective for major depressive disorder?. Medwave.

[24] Zheng J., Baird D., Borges M.-C., Bowden J., Hemani G., Haycock P., Evans D.M., Smith G.D. (2017). Recent Developments in Mendelian Randomization Studies. Curr. Epidemiol. Rep..

[25] Bowden J., Holmes M.V. (2019). Meta-analysis and Mendelian randomization: A review. Res. Synth. Methods.

[26] Sekula P., Del Greco M.F., Pattaro C., Köttgen A. (2016). Mendelian Randomization as an Approach to Assess Causality Using Observational Data. J. Am. Soc. Nephrol. JASN.

[27] Jin T., Huang W., Pang Q., He Z., Yuan L., Zhang H., Xing D., Guo S., Zhang T. (2024). Inferring the genetic effects of serum homocysteine and vitamin B levels on autism spectral disorder through Mendelian randomization. Eur. J. Nutr..

[28] Skrivankova V.W., Richmond R.C., Woolf B.A., Yarmolinsky J., Davies N.M., Swanson S.A., VanderWeele T.J., Higgins J.P.T., Timpson N.J., Richards J.B. (2021). Strengthening the Reporting of Observational Studies in Epidemiology Using Mendelian Randomization: The STROBE-MR Statement. JAMA.

[29] Van Meurs J.B., Pare G., Schwartz S.M., Hazra A., Tanaka T., Vermeulen S.H., Cotlarciuc I., Yuan X., Mälarstig A., Ahmadi K.R. (2013). Common genetic loci influencing plasma homocysteine concentrations and their effect on risk of coronary artery disease. Am. J. Clin. Nutr..

[30] Burgess S., Thompson S.G., CRP CHD Genetics Collaboration (2011). Avoiding bias from weak instruments in Mendelian randomization studies. Int. J. Epidemiol..

[31] Burgess S., Scott R.A., Timpson N.J., Smith G.D., Thompson S.G., EPIC-InterAct Consortium (2015). Using published data in Mendelian randomization: A blueprint for efficient identification of causal risk factors. Eur. J. Epidemiol..

[32] Bowden J., Smith G.D., Haycock P.C., Burgess S. (2016). Consistent Estimation in Mendelian Randomization with Some Invalid Instruments Using a Weighted Median Estimator. Genet. Epidemiol..

[33] Bowden J., Davey Smith G., Burgess S. (2015). Mendelian randomization with invalid instruments: Effect estimation and bias detection through Egger regression. Int. J. Epidemiol..

[34] Burgess S., Butterworth A., Thompson S.G. (2013). Mendelian randomization analysis with multiple genetic variants using summarized data. Genet. Epidemiol..

[35] Verbanck M., Chen C.-Y., Neale B., Do R. (2018). Detection of widespread horizontal pleiotropy in causal relationships inferred from Mendelian randomization between complex traits and diseases. Nat. Genet..

[36] Curtin F., Schulz P. (1998). Multiple correlations and bonferroni’s correction. Biol. Psychiatry.

[37] Yuan S., Mason A.M., Carter P., Burgess S., Larsson S.C. (2021). Homocysteine, B vitamins, and cardiovascular disease: A Mendelian randomization study. BMC Med..

[38] O’Leary F., Samman S. (2010). Vitamin B12 in health and disease. Nutrients.

[39] Yoon S.I., Moon H.R., Lee S.R., Zhang J., Lee S., Cho J.A. (2023). Nutrient Inadequacy in Korean Young Adults with Depression: A Case Control Study. Nutrients.

[40] Yadav A., Bharti A., Tevatia M., Prakash J., Bajaj S. (2023). Are vitamin D, B12, and folate deficiency associated with depressive disorder? A case-control study. Ind. Psychiatry J..

[41] Vahid F., Rahmani W., Davoodi S.H., Bohn T. (2023). Mental Health Conditions, Including Depression and Stress, Are Associated with Increased Odds of Gastric Cancer-Insights into the Role of Diet: A Case-Control Study. Nutrients.

[42] Esnafoglu E., Ozturan D.D. (2020). The relationship of severity of depression with homocysteine, folate, vitamin B12, and vitamin D levels in children and adolescents. Child Adolesc. Ment. Health.

[43] Bottiglieri T. (2005). Homocysteine and folate metabolism in depression. Prog. Neuro-Psychopharmacol. Biol. Psychiatry.

[44] Folstein M., Liu T., Peter I., Buel J., Arsenault L., Scott T., Qiu W.W. (2007). The homocysteine hypothesis of depression. Am. J. Psychiatry.

[45] Bhatia P., Singh N. (2015). Homocysteine excess: Delineating the possible mechanism of neurotoxicity and depression. Fundam. Clin. Pharmacol..

[46] Sahu P., Thippeswamy H., Chaturvedi S.K. (2022). Neuropsychiatric manifestations in vitamin B12 deficiency. Vitam. Horm..

[47] Tan Y., Zhou L., Huang J., Chen X., Wu Y., Song X., Wang J., Hu H., Yang Q. (2023). Vitamin B12, Folate, Homocysteine, Inflammatory Mediators (Interleukin-6, Tumor Necrosis Factor-α and C-Reactive Protein) Levels in Adolescents with Anxiety or Depressive Symptoms. Neuropsychiatr. Dis. Treat..

[48] Mishra G.D., McNaughton S.A., O’connell M.A., Prynne C.J., Kuh D. (2009). Intake of B vitamins in childhood and adult life in relation to psychological distress among women in a British birth cohort. Public Health Nutr..

[49] Mozaffari H., Mofrad M.D., Surkan P.J., Askari M., Azadbakht L. (2021). Associations between dietary intake of B-vitamins and psychological disorders among Iranian women: A cross-sectional study. Public Health Nutr..

[50] Marazziti D., Mangiapane P., Carbone M.G., Morana F., Arone A., Massa L., Palermo S., Violi M., Bertini G., Massoni L. (2023). Decreased Levels of Vitamin D in Bipolar Patients. Life.

[51] Lachner C., Martin C., John D., Nekkalapu S., Sasan A., Steinle N., Regenold W.T. (2014). Older adult psychiatric inpatients with non-cognitive disorders should be screened for vitamin B12 deficiency. J. Nutr. Health Aging.

[52] Andrès E., Serraj K., Zhu J., Vermorken A. (2013). The pathophysiology of elevated vitamin B12 in clinical practice. QJM Mon. J. Assoc. Physicians.

[53] Shipton M.J., Thachil J. (2015). Vitamin B12 deficiency—A 21st century perspective. Clin. Med..

[54] Arendt J.F.B., Nexo E. (2012). Cobalamin related parameters and disease patterns in patients with increased serum cobalamin levels. PLoS ONE.

[55] Raghavan R., Riley A.W., Volk H., Caruso D., Hironaka L., Sices L., Hong X., Wang G., Ji Y., Wang X. (2018). Maternal Multivitamin Intake, Plasma Folate and Vitamin B(12) Levels and Autism Spectrum Disorder Risk in Offspring. Paediatr. Perinat. Epidemiol..

[56] Hope S., Nærland T., Høiland A.L., Torske T., Malt E., Abrahamsen T., Nerhus M., Wedervang-Resell K., Lonning V., Andreassen O.A. (2020). Higher vitamin B12 levels in neurodevelopmental disorders than in healthy controls and schizophrenia: A comparison among participants between 2 and 53 years. FASEB J..

[57] Shibata K., Hirose J., Fukuwatari T. (2014). Relationship between Urinary Concentrations of Nine Water-soluble Vitamins and their Vitamin Intakes in Japanese Adult Males. Nutr. Metab. Insights.

[58] Andrew T., Gill R., Gillham-Nasenya I., Ahmadi K.R. (2013). Unravelling the basis of variability in cobalamin levels in the general population. Br. J. Nutr..

[59] Tanaka T., Scheet P., Giusti B., Bandinelli S., Piras M.G., Usala G., Lai S., Mulas A., Corsi A.M., Vestrini A. (2009). Genome-wide association study of vitamin B6, vitamin B12, folate, and homocysteine blood concentrations. Am. J. Hum. Genet..

[60] Grarup N., Sulem P., Sandholt C.H., Thorleifsson G., Ahluwalia T.S., Steinthorsdottir V., Bjarnason H., Gudbjartsson D.F., Magnusson O.T., Sparsø T. (2013). Genetic architecture of vitamin B12 and folate levels uncovered applying deeply sequenced large datasets. PLoS Genet..

