# Supplementary material for: Exploring the Association between Serum B Vitamins, Homocysteine and Mental Disorders: Insights from Mendelian Randomization
Source: Nutrients. 2024 Jun 21;16(13):1986. doi: 10.3390/nu16131986 (PMC11243542; doi:10.3390/nu16131986)
Supplement: Supplementary file 1 [file nutrients-16-01986-s001.zip › nutrients-3048867-supplementary.pdf]

Supplementary Materials

Supplementary Table S1. *STROBE-MR checklist of the present study.*

Supplementary Table S2. *Information on instrumental variables.*

Supplementary Table S3. *Heterogeneity of MR analysis for serum levels of folate, vitamin B6, vitamin B12, homocysteine and mental disorders risk.*

Supplementary Table S4. *Associations of genetic prediction of serum levels of folate, vitamin B6, vitamin B12 and homocysteine with mental disorders risk in the MR-Egger analysis.*

Supplementary Table S5. *Associations of genetic prediction of serum levels of folate, vitamin B6, vitamin B12 and homocysteine with mental disorders risk in the MR-PRESSO analysis.*

Supplementary Table S6. *Heterogeneity of MR analysis for serum levels of folate, homocysteine and depression risk after excluding the outlier SNPs.*

Supplementary Table S7. *MR analysis for serum levels of folate, homocysteine and depression risk after excluding the outlier SNPs.*

Supplementary Figure S1. *Scatter plot of the association between vitamin B12 and mental disorders. (A)anxiety disorders; (B) Bipolar affective disorders.*

Supplementary Figure S2. *Funnel plot on vitamin B12 and mental disorders. (A)anxiety disorders; (B) Bipolar affective disorders.*

Supplementary Figure S3. *Leave-one-out plots of vitamin B12 and mental disorders. (A)anxiety disorders; (B) Bipolar affective disorders.*

Supplementary Table S1. STROBE-MR checklist of the present study

No.	Section	Checklist item	Manuscript section and paragraph
1	Title and abstract	Indicate MR as the study's design in the title and the abstract as a main purpose of the study	Title, Abstract
2	Background	Explain the scientific background and rationale for the reported study. Explain the exposure and a plausible potential causal relationship between exposure and outcome. Justify why MR is a helpful method to address the study question.	Introduction: P1-P3
3	Objectives	State specific objectives clearly, including prespecified causal hypotheses. State that MR is a method that intends to estimate causal effects.	Introduction: P4
4a	Study design and data sources	Setting: Describe the study design (two-sample MR) and the underlying population . Describe the setting, locations, and relevant dates, including periods of recruitment, exposure, follow-up, and data collection.	Materials and methods: Study design, Figure 1, Table S2
4b		Participants: Report the eligibility criteria and the sources and methods of selection of participants. Report the sample size and whether any power or sample size calculations were carried out prior to the main analysis.	Materials and methods: Data Sources, Table 1
4c		Describe measurement, quality control, and selection of genetic variants.	Materials and methods: Selection of Instrumental Variables
4d		For each exposure, outcome, and other relevant variables, describe methods of assessment and diagnostic criteria for diseases.	Materials and methods: Data Sources, Table 1, Figure 1
4e		Provide details of ethics committee approval and participant informed consent, if relevant.	Materials and methods: Data Sources

5	Assumptions	Explicitly state the 3 core instrumental variable (IV) assumptions for the main analysis (relevance, independence, and exclusion restriction), as well assumptions for any additional or sensitivity analysis.	Materials and methods: Study design, Figure 1
6a	Statistical methods:	Describe how quantitative variables were handled in the analyses.	Materials and methods: Statistical analysis
6b		Describe how genetic variants were handled in the analyses and, if applicable, how their weights were selected	Materials and methods: Statistical analysis
6c		Describe the MR estimator and related statistics. Detail the included covariates and, in case of 2-sample MR, whether the same covariate set was used for adjustment in the 2 samples.	Materials and methods: Statistical analysis
6d		Explain how missing data were addressed.	N/A
6e		Indicate how multiple testing was addressed (Bonferroni correction)	Materials and methods: Statistical analysis
7	Assessment of assumptions	Describe any methods or prior knowledge used to assess the assumptions or justify their validity	Materials and methods: Statistical analysis
8	Sensitivity analyses and additional analyses	Describe any sensitivity analyses or additional analyses performed (eg, comparison of effect estimates from different approaches, independent replication, bias analytic techniques, validation of instruments, simulations).	Materials and methods: Statistical analysis
9a	Software and preregistration	Name statistical software and package(s), including version and settings used.	Materials and methods: Statistical analysis
9b		State whether the study protocol and details were preregistered (as well as when and where).	N/A

10a	Descriptive data	Report the numbers of individuals at each stage of included studies and reasons for exclusion. Use of a flow diagram.	N/A
10b		Report summary statistics for phenotypic exposure , outcomes, and other relevant variables (eg, means, SDs, proportions).	Table S2
10c		If the data sources include meta-analyses of previous studies, provide the assessments of heterogeneity across these studies.	N/A
10d		For 2-sample MR:	
		i. Provide justification of the similarity of the genetic variant–exposure associations between the exposure and outcome samples.	Materials and methods: Selection of Instrumental Variables
		ii. Provide information on the number of individuals who overlap between the exposure and outcome studies.	N/A
11a	Main results	Report the associations between genetic variant and exposure and between genetic variant and outcome, preferably on an interpretable scale.	Results: Mendelian Randomization Estimates, Figure 2-5, Figure S1-S3
11b		Report MR estimates of the relationship between exposure and outcome and the measures of uncertainty from the MR analysis, on an interpretable scale, such as odds ratio or relative risk per SD difference.	Results: Mendelian Randomization Estimates, Figure 2-5, Figure S1-S3
11c		If relevant, consider translating estimates of relative risk into absolute risk for a meaningful time period.	N/A
11d		Consider plots to visualize results (eg, forest plot, scatterplot of associations between genetic variants and outcome vs between genetic variants and exposure).	Figure 2-5, Figure S1-S3

12a	Assessment of assumptions	Report the assessment of the validity of the assumptions by removing confounders-related SNPs.	N/A
12b		Report any additional statistics (eg, assessments of heterogeneity across genetic variants, such as I^2, Q statistic).	Results: Evaluation of Mendelian Randomization Assumptions
13a	Sensitivity analyses and additional analyses	Report any sensitivity analyses to assess the robustness of the main results to violations of the assumptions.	Results: Evaluation of Mendelian Randomization Assumptions
13b		Report results from other sensitivity analyses or additional analyses	Results: Evaluation of Mendelian Randomization Assumptions, Table S3-S7
13c		Report any assessment of the direction of the causal relationship.	Results: Mendelian Randomization Estimates, Figure 2-5, Figure S1-S3
13d		When relevant, report and compare with estimates from other RCTs and meta-analyses.	N/A
13e		Consider additional plots to visualize results.	Figure 2-5, Figure S1-S3
14	Key results	Summarize key results with reference to study objectives.	Discussion: P1
15	Limitations	Discuss limitations of the study, taking into account the validity of the IV assumptions, other sources of potential bias, and imprecision. Discuss both direction and magnitude of any potential bias and any efforts to address them.	Discussion: P7
16a	Interpretation	Meaning: Give a cautious overall interpretation of results in the context of their limitations and in comparison with other studies.	Discussion: P2
16b		Mechanism: Discuss underlying biological mechanisms that could drive a potential causal relationship between the investigated exposure and the	Discussion: P3

		outcome, and whether the gene-environment equivalence assumption is reasonable. Use causal language carefully, clarifying that IV estimates may provide causal effects only under certain assumptions.	
16c		Clinical relevance: Discuss whether the results have clinical or public policy relevance, and to what extent they inform effect sizes of possible interventions.	Discussion: P5
17		Discuss the generalizability of the study results (a) to other populations, (b) across other exposure periods/timings, and (c) across other levels of exposure.	Discussion: P4
18	Funding	Describe sources of funding and the role of funders in the present study.	N/A
19	Data and data sharing	Provide the data used to perform all analyses or report where and how the data can be accessed, and reference these sources in the article.	Table 1
20	Conflicts of interest	All authors should declare all potential conflicts of interest.	Declaration

Supplementary Table S2. Information on instrumental variables.

Exposure	SNP	Nearest Gene	Chr	Position	EA	OA	β	SE	EAF	*p* value	R^2*	F - statistic**
Folate												
1	rs78074774	LINC01317	2	34515489	T	C	0.0601	0.0132	0.0447	4.90E-06	3.09E-04	20.07
2	rs3772928	GAP43	3	115406478	T	C	-0.0273	0.0055	0.5754	8.20E-07	3.16E-04	20.51
3	rs76630415	DGKB	7	14144445	T	G	-0.0374	0.0067	0.2120	2.40E-08	3.63E-04	23.60
4	rs2449166	CSMD1	8	3463535	T	C	0.0252	0.0055	0.4714	4.00E-06	4.67E-04	30.37
5	rs45442894	CUBN	10	16870693	A	C	0.1096	0.0240	0.0141	5.00E-06	3.17E-04	20.61
6	rs7074988	GRID1	10	87848038	A	G	-0.0513	0.0111	0.0637	4.00E-06	3.34E-04	21.68
7	rs1502443	LOC124903724	16	77060066	G	C	0.0259	0.0056	0.6300	4.30E-06	3.14E-04	20.39
8	rs16956822	CD68	17	7499349	G	A	-0.0793	0.0172	0.0262	3.90E-06	3.73E-04	24.24
9	rs148031795	COLEC12	18	482310	T	C	0.1044	0.0224	0.0150	3.10E-06	3.13E-04	20.32
10	rs8085166	LPIN2	18	3016615	G	A	0.0278	0.0058	0.6769	1.70E-06	3.20E-04	20.82
11	rs79975477	ESF1	20	13683165	T	C	0.0731	0.0156	0.0310	2.80E-06	3.22E-04	20.95
12	rs76802001	DUSP18	22	31013399	G	A	-0.0677	0.0148	0.0362	4.60E-06	3.38E-04	21.99
13	rs79748722	ATXN10	22	46147495	C	T	-0.0757	0.0165	0.0280	4.40E-06	3.22E-04	20.90
VitaminB6												
1	rs188211816	NA	1	172810049	A	G	-0.0786	0.0163	0.0295	1.40E-06	3.54E-04	22.99
2	rs155599	CYTIP	2	158307375	C	T	0.0343	0.0060	0.7052	1.00E-08	4.88E-04	27.17
3	rs3772928	GAP43	3	115406478	C	T	-0.0292	0.0055	0.5754	1.30E-07	4.18E-04	21.99

4	rs141933624	CCSER1	4	91118956	A	G	-0.0897	0.0193	0.0215	3.30E-06	3.38E-04	22.82
5	rs183178622	BDH2	4	103988054	T	C	-0.0989	0.0207	0.0183	1.70E-06	3.51E-04	21.70
6	rs77806858	MFAP3	5	153476869	C	T	-0.0505	0.0106	0.0704	1.90E-06	3.34E-04	20.12
7	rs12198456	NA	6	120371988	T	C	0.0920	0.0197	0.0187	3.10E-06	3.10E-04	22.79
8	rs74640671	GFRA2	8	21550838	T	C	-0.1272	0.0274	0.0110	3.40E-06	3.51E-04	23.48
9	rs12412051	LOC105376350	10	2601269	C	G	0.0709	0.0150	0.0344	2.20E-06	3.33E-04	20.77
10	rs12226112	SPON1	11	14163360	T	G	0.0283	0.0057	0.3414	7.50E-07	3.61E-04	20.36
11	rs361294	PDGFD	11	103993635	C	A	-0.0274	0.0060	0.6913	4.30E-06	3.20E-04	21.77
12	rs9560457	NA	13	90476979	T	C	0.0255	0.0055	0.4042	4.10E-06	3.13E-04	21.16
13	rs34938615	OR4E2	14	22113999	G	A	-0.1220	0.0264	0.0114	3.90E-06	3.35E-04	20.94
14	rs10138490	DIO2-AS1	14	80883798	C	T	-0.0535	0.0114	0.0605	2.80E-06	3.26E-04	21.50
15	rs7205927	NA	16	48911591	C	A	-0.0258	0.0055	0.4119	3.20E-06	3.22E-04	22.99
16	rs3745438	CDC42EP51	19	54972918	C	T	-0.0713	0.0156	0.0337	4.90E-06	3.31E-04	27.17
17	rs67450584	EFCAB6	22	44202370	T	C	0.0367	0.0075	0.1580	8.60E-07	3.58E-04	22.82
Vitamin B12												
1	rs10924919	C1orf229	1	247272298	T	C	-0.0286	0.0056	0.3945	3.90E-07	3.92E-04	25.45
2	rs67568068	ATP10B	5	160155731	C	T	-0.0317	0.0066	0.2127	1.80E-06	3.37E-04	21.93
3	rs148901823	AOAH	7	36745068	G	A	-0.0486	0.0101	0.0807	1.30E-06	3.51E-04	22.81
4	rs193228340	NA	8	27008230	C	T	0.1563	0.0340	0.0071	4.30E-06	3.46E-04	22.49
5	rs12776611	C10orf35	10	71424622	A	G	-0.0878	0.0189	0.0221	3.40E-06	3.34E-04	21.68
6	rs10749205	CCDC172	10	118046389	C	T	0.0316	0.0068	0.7996	3.90E-06	3.19E-04	20.75
7	rs61994378	NA	14	106827305	C	T	0.0935	0.0198	0.0278	2.30E-06	4.72E-04	30.69
8	rs388561	LINC01643	22	34612149	C	T	0.0403	0.0085	0.8807	2.50E-06	3.41E-04	22.16

Homocysteine												
1	rs2275565	ACTN2	1	236885376	T	G	-0.0542	0.009	0.21	1.72E-09	9.74E-04	43.07
2	rs4660306	HECTD3	1	45513003	T	C	0.0435	0.007	0.33	5.16E-10	8.37E-04	36.97
3	rs9369898	CENPQ	6	49414480	A	G	0.0449	0.007	0.62	1.41E-10	9.50E-04	41.98
4	rs548987	SLC17A1	6	25869143	C	G	0.0597	0.01	0.13	2.37E-09	8.06E-04	35.62
5	rs42648	CDK14	7	90348446	A	G	-0.0395	0.007	0.4	1.67E-08	7.49E-04	33.09
6	rs12780845	CUBN	10	17181245	A	G	0.0529	0.009	0.65	4.16E-09	1.27E-03	56.28
7	rs1801222	CUBN	10	17114152	A	G	0.0453	0.007	0.34	9.71E-11	9.21E-04	40.69
8	rs7130284	FOLH1B	11	89415204	T	C	-0.1242	0.013	0.07	1.25E-21	2.01E-03	88.84
9	rs2251468	COQ5	12	120967323	A	C	-0.0512	0.007	0.65	2.59E-13	1.19E-03	52.72
10	rs154657	CPNE7	16	89641688	A	G	0.0963	0.007	0.47	4.61E-43	4.62E-03	204.90
11	rs1801133	DNAH9	17	11796321	A	G	0.1583	0.007	0.34	3.14E-113	1.12E-02	502.12
12	rs838133	CARD8	19	48756272	A	G	0.0422	0.007	0.45	1.65E-09	8.82E-04	38.95

SNP, single nucleotide polymorphisms; EA, effect allele; OA, other allele; SE, standard error; EAF: effect allele frequency; *R^2 =2×EAF×(1-EAF) ×$(\beta)^2$; **F - statistic=R^2×(N-2) / (1-R^2)

NOTE: R^2(The variance): the proportion of exposed variability explained by individual genetic instrument; EAF: the effect allele frequency; β: the estimated effect of SNP; N: the sample size of the exposure of GWAS.

Supplementary Table S3. Heterogeneity of MR analysis for serum levels of folate, vitamin B6, vitamin B12, homocysteine and mental disorders risk.

Exposure	Outcome	Method	Q	Q_df	Q_*p* value
Folate	Depression	MR-Egger	23.697	11	0.014
		Inverse variance weighted	25.914	12	0.011
	Anxiety disorders	MR-Egger	6.803	11	0.815
		Inverse variance weighted	13.807	12	0.313
	Bipolar affective disorders	MR-Egger	18.932	11	0.062
		Inverse variance weighted	19.738	12	0.072
	Obsessive-compulsive disorder	MR-Egger	14.531	11	0.205
		Inverse variance weighted	14.541	12	0.267
	Schizophrenia	MR-Egger	6.268	11	0.855
		Inverse variance weighted	6.277	12	0.901
Vitamin B6	Depression	MR-Egger	20.606	15	0.150
		Inverse variance weighted	22.138	16	0.139
	Anxiety disorders	MR-Egger	12.064	15	0.674
		Inverse variance weighted	19.466	16	0.245
	Bipolar affective disorders	MR-Egger	5.936	15	0.981
		Inverse variance weighted	8.245	16	0.941
	Obsessive-compulsive disorder	MR-Egger	17.705	15	0.279
		Inverse variance weighted	18.094	16	0.318
	Schizophrenia	MR-Egger	8.127	15	0.919
		Inverse variance weighted	9.461	16	0.893
Vitamin B12	Depression	MR-Egger	5.218	6	0.516
		Inverse variance weighted	5.754	7	0.569
	Anxiety disorders	MR-Egger	4.709	6	0.582

		Inverse variance weighted	5.108	7	0.647
	Bipolar affective disorders	MR-Egger	2.315	6	0.889
		Inverse variance weighted	2.316	7	0.940
	Obsessive-compulsive disorder	MR-Egger	4.315	6	0.634
		Inverse variance weighted	4.491	7	0.722
	Schizophrenia	MR-Egger	7.005	6	0.320
		Inverse variance weighted	7.023	7	0.426
Homocysteine	Depression	MR-Egger	26.777	10	0.003
		Inverse variance weighted	26.940	11	0.005
	Anxiety disorders	MR-Egger	12.498	10	0.253
		Inverse variance weighted	13.378	11	0.269
	Bipolar affective disorders	MR-Egger	17.351	10	0.067
		Inverse variance weighted	17.951	11	0.083
	Obsessive-compulsive disorder	MR-Egger	16.455	10	0.087
		Inverse variance weighted	17.154	11	0.103
	Schizophrenia	MR-Egger	15.187	10	0.125
		Inverse variance weighted	15.576	11	0.158

Supplementary Table S4. Associations of genetic prediction of serum levels of folate, vitamin B6, vitamin B12 and homocysteine with mental disorders risk in the MR-Egger analysis.

Exposure	Outcome	SNPs	Egger_intercept	SE	*p*
Folate	Depression	13	-0.009	0.009	0.332
	Anxiety disorders	13	-0.026	0.010	0.023
	Bipolar affective disorders	13	-0.014	0.021	0.508
	Obsessive-compulsive disorder	13	0.003	0.032	0.931
	Schizophrenia	13	-0.002	0.023	0.924
Vitamin B6	Depression	17	0.008	0.007	0.308
	Anxiety disorders	17	0.025	0.009	0.016
	Bipolar affective disorders	17	0.023	0.015	0.149
	Obsessive-compulsive disorder	17	0.017	0.029	0.574
	Schizophrenia	17	0.026	0.022	0.266
Vitamin B12	Depression	8	-0.007	0.010	0.491
	Anxiety disorders	8	-0.010	0.015	0.551
	Bipolar affective disorders	8	0.001	0.025	0.978
	Obsessive-compulsive disorder	8	0.018	0.044	0.689
	Schizophrenia	8	-0.005	0.040	0.905
Homocysteine	Depression	12	0.002	0.008	0.810
	Anxiety disorders	12	-0.007	0.008	0.421
	Bipolar affective disorders	12	0.009	0.016	0.569
	Obsessive-compulsive disorder	12	0.016	0.029	0.585
	Schizophrenia	12	-0.011	0.022	0.624

Supplementary Table S5. Associations of genetic prediction of serum levels of folate, vitamin B6, vitamin B12 and homocysteine with mental disorders risk in the MR-PRESSO analysis.

Exposure	Outcome	SNPs	Outliers		Causal Estimate	SD	T-stat	*p* value	Global *p* value
Folate	Depression	13	2	Raw	0.051	0.103	0.492	0.631	0.018
				Outlier-corrected	0.119	0.069	1.725	0.112	
	Anxiety disorders	13	0		0.123	0.114	1.073	0.304	0.322
	Bipolar affective disorders	13	0		0.063	0.222	0.286	0.780	0.110
	Obsessive-compulsive disorder	13	0		0.418	0.335	1.246	0.236	0.277
	Schizophrenia	13	0		-0.268	0.183	-1.465	0.169	0.890
Vitamin B6	Depression	17	0		0.076	0.077	0.977	0.343	0.142
	Anxiety disorders	17	0		-0.054	0.111	-0.491	0.630	0.247
	Bipolar affective disorders	17	0		0.070	0.118	0.599	0.557	0.937
	Obsessive-compulsive disorder	17	0		-0.241	0.306	-0.788	0.442	0.319
	Schizophrenia	17	0		-0.159	0.184	-0.864	0.400	0.892
Vitamin B12	Depression	8	0		0.078	0.087	0.900	0.398	0.642
	Anxiety disorders	8	0		0.291	0.125	2.337	0.052	0.674
	Bipolar affective disorders	8	0		0.617	0.137	4.507	0.003	0.937
	Obsessive-compulsive disorder	8	0		0.069	0.335	0.205	0.844	0.699
	Schizophrenia	8	0		0.117	0.352	0.331	0.750	0.466
Homocysteine	Depression	12	9	Raw	0.031	0.050	0.621	0.547	0.014
				Outlier-corrected	0.048	0.039	1.245	0.242	

Anxiety disorders	12	0	-0.026	0.054	-0.481	0.640	0.322
Bipolar affective disorders	12	0	-0.054	0.101	-0.534	0.604	0.132
Obsessive-compulsive disorder	12	0	0.110	0.174	0.634	0.539	0.102
Schizophrenia	12	0	0.048	0.137	0.350	0.733	0.148

Supplementary Table S6. Heterogeneity of MR analysis for serum levels of folate, homocysteine and depression risk after excluding the outlier SNPs.

Exposure	Outcome	SNPs	T-State	*p* for MR-PRESSO	Egger_intercept	*p* for pleiotropy	Cochrane's Q	*p* for Cochrane's Q
Folate	Depression	12	1.725	0.549	0.001	0.882	10.123	0.519
Homocysteine	Depression	11	1.245	0.179	0.004	0.536	14.364	0.157

Supplementary Table S7. MR analysis for serum levels of folate, homocysteine and depression risk after excluding the outlier SNPs.

Exposure	Outcome	SNPs	Method	*p*	OR(95%CI)
Folate	Depression	12	MR Egger	0.530	1.104(0.820-1.486)
			Weighted median	0.472	1.076(0.882-1.313)
			Inverse variance weighted	0.098	1.126(0.978-1.297)
Homocysteine	Depression	11	MR Egger	0.986	1.001(0.852-1.178)
			Weighted median	0.997	1.000(0.915-1.093)
			Inverse variance weighted	0.213	1.049(0.973-1.132)

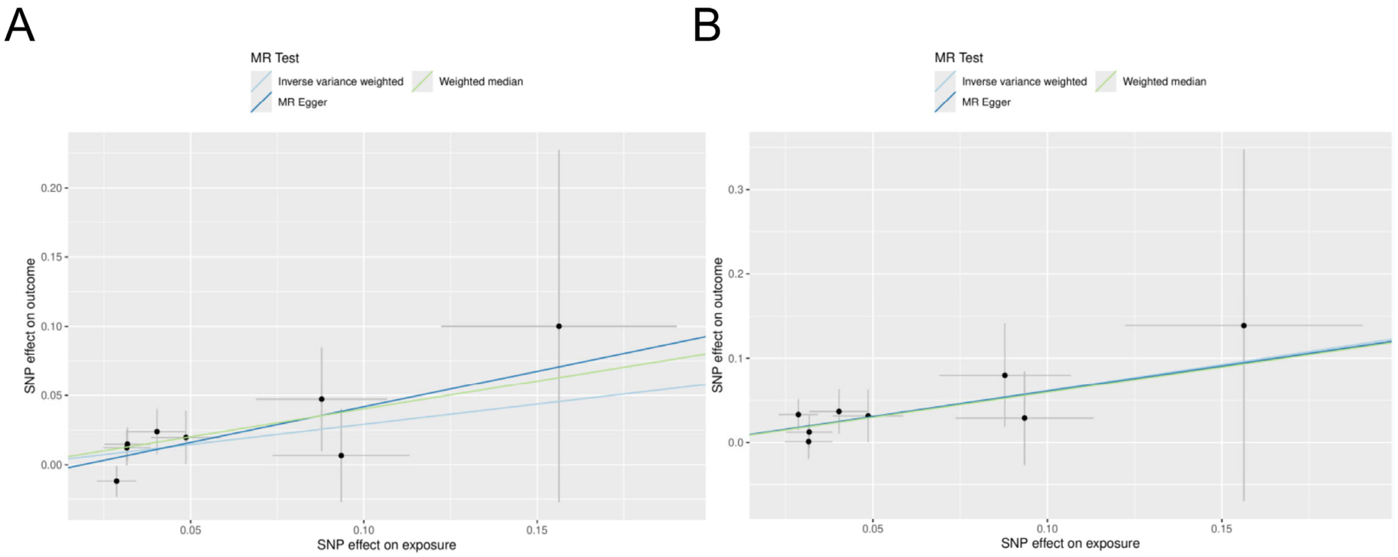

Supplementary Figure S1. Scatter plot of the association between vitamin B12 and mental disorders. (A)anxiety disorders(B) Bipolar affective disorders. Three lines reveal the estimated effect sizes by MR methods (inverse-variance weighted, MR-Egger and weighted median).

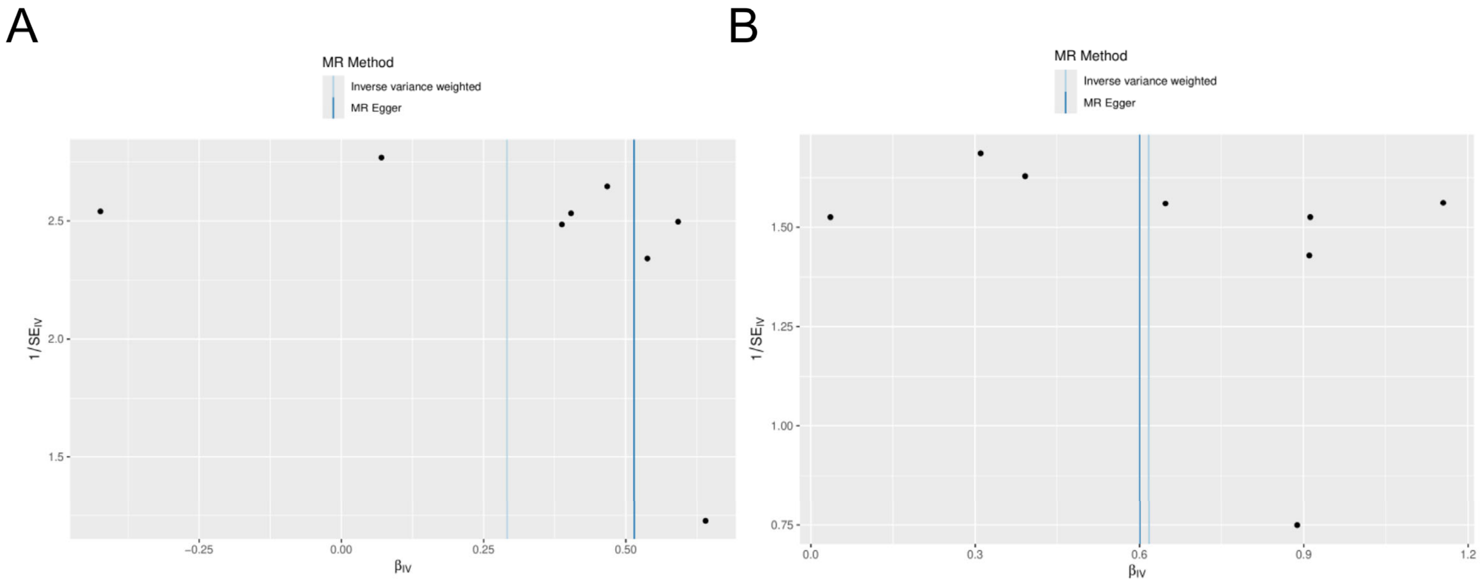

Supplementary Figure S2. Funnel plot on vitamin B12 and mental disorders. (A)anxiety disorders; (B) Bipolar affective disorders. Figure 2B is symmetric, while Figure 2A may demonstrate asymmetry. Given the limited number of SNPs for vitamin B12 (n=8), assessing symmetry becomes challenging due to insufficient test power to distinguish chance from real asymmetry. Despite these challenges, we prioritized rigorous quantitative sensitivity analyses in our study, including F-statistics, Cochran's Q test, MR-Egger's intercept, and MR-PRESSO. Our analysis found no significant detection of weak IVs, heterogeneity, or pleiotropy. Thus, we maintain confidence that our findings do not definitively confirm the existence of pleiotropy and heterogeneity.

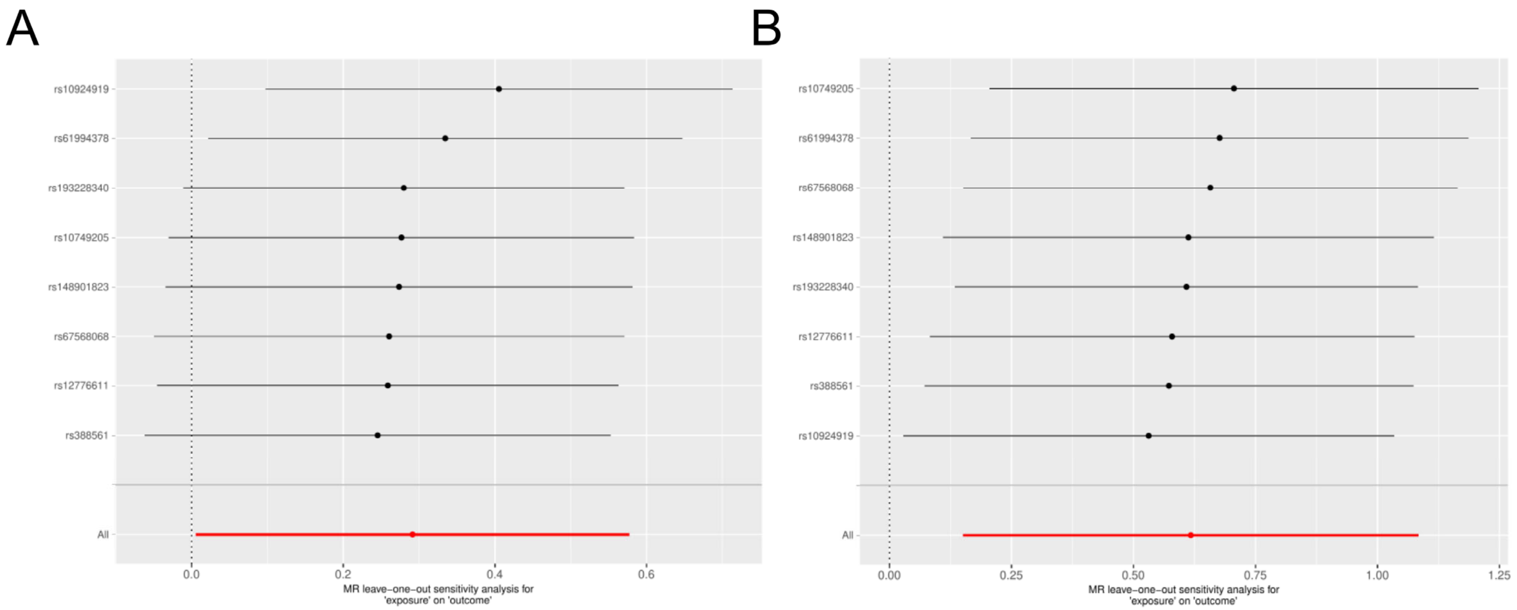

Supplementary Figure S3. Leave-one-out plots of vitamin B12 and mental disorders. (A)anxiety disorders; (B) Bipolar affective disorders.
